# An analysis of hospital preparedness capacity for public health emergency in four regions of China: Beijing, Shandong, Guangxi, and Hainan

**DOI:** 10.1186/1471-2458-8-319

**Published:** 2008-09-20

**Authors:** Xingming Li, Jianshi Huang, Hui Zhang

**Affiliations:** 1 Department of Epidemiology, Institute of Basic Medical Sciences, Chinese Academy of Medical Sciences; School of Basic Medicine, Peking Union Medical College, No. 5 Dongdan Santiao, Beijing 100005, PR China; 2 Chinese Centers for Diseases Control and Prevention, Beijng, PR China

## Abstract

**Background:**

Hospital preparedness is critical for the early detection and management of public health emergency (PHE). Understanding the current status of PHE preparedness is the first step in planning to enhance hospitals' capacities for emergency response. The objective of this study is to understand the current status of hospital PHE preparedness in China.

**Methods:**

Four hundred hospitals in four city and provinces of China were surveyed using a standardized questionnaire. Data related to hospital demographic data; PHE preparation; response to PHE in community; stockpiles of drugs and materials; detection and identification of PHE; procedures for medical treatment; laboratory diagnosis and management; staff training; and risk communication were collected and analyzed.

**Results:**

Valid responses were received from 318 (79.5%) of the 400 hospitals surveyed. Of the valid responses, 264 (85.2%) hospitals had emergency plans; 93.3% had command centres and personnel for PHE; 22.9% included community organisations during the training for PHE; 97.4% could transport needed medical staff to a PHE; 53.1% had evaluated stockpiles of drugs; 61.5% had evaluated their supply systems; 55.5% had developed surveillance systems; and 74.6% could monitor the abnormity(See in appendix). Physicians in 80.2% of the analyzed hospitals reported up-to-date knowledge of their institution's PHE protocol. Of the 318 respondents, 97.4% followed strict laboratory regulations, however, only about 33.5% had protocols for suspected samples. Furthermore, only 59.0% could isolate and identify salmonella and staphylococcus and less than 5% could isolate and identify human H5N1 avian flu and SARS. Staff training or drill programs were reported in 94.5% of the institutions; 50.3% periodically assessed the efficacy of staff training; 45% had experts to provide psychological counselling; 12.1% had provided training for their medical staff to assess PHE-related stress. All of the above capacities related to the demographic characteristics of hospitals and will be discussed in-depth in this paper.

**Conclusion:**

Our survey suggested that, at the time of the survey, hospital preparedness for PHE in China was at an early stage of development. Comprehensive measures should be taken to enhance hospital capacity in the prevention and management of PHE.

## Background

Public health emergency (PHE) is an event or events that cause or may cause harm to the health of a community or nation [1]. To prevent and/or minimize the harm caused by PHE, early detection and management are necessary. As hospitals are the main location for PHE surveillance and treatment, their preparedness is critical for PHE's early detection and management [2]. Evaluating the current status of PHE preparedness within the hospital system is the first step in improving a nation's preparedness for a PHE. Yet, there is no national data on China's hospital PHE preparedness capacity aside from two studies that addressed the issues at local level [3,4]. To understand the current status of hospital PHE preparedness in China, a sample survey of hospitals in four representative city/provinces were conducted between November 2004 and March 2005.

## Methods

### Study design

The survey used a cross-sectional study design to survey hospitals in different regions of China. Respondents were all secondary and tertiary hospitals(the detail of hospital classification see in appendix) in the city of Beijing and provinces of Shandong, Guangxi, and Hainan. The selection of hospitals in these four regions is intended to represent a variety of regional economic status. Broadly speaking, Beijing and Shandong are economically well developed, Hainan moderately developed, and Guangxi developing [5]. According to the Hospital Classification Method issued by the National Bureau of Statistics of China, the surveyed hospitals included general hospitals, hospitals of traditional Chinese medicine (TCM), hospitals of integrated traditional Chinese medicine and western medicine (TCM-WM), specialized hospitals, community health center, and medical emergency center (the definition of community health center and medical emergency center see in appendix) [6]. Four hundred secondary and tertiary hospitals were surveyed. The study was approved by the Institutional Review Board (IRB) of the School of Basic Medicine, Peking Union Medical College in Beijing, China.

### Survey instruments

Based on a literature and government document review, a detailed methodological approach for research framework and questionnaire development was followed to inform the development of this study [3]. An indicator system framework was created and questionnaire designed based on the framework. The questionnaire consists of 17 sections and 192 items. The questionnaire and the survey protocol (including field work manual and quality control procedures) were tested by a pilot study. For the purpose of this study, we analyzed the data focused on the following nine areas of interest: (1) hospital's demographic data (including region, SARS crisis experience, teaching function, hospital type, and number of medical staff in related departments); (2) hospital PHE preparation (emergency plans, response initiating time, accessibility, and revision and implementation of emergency plan); (3) response to a community PHE (cooperation with local organizations, relationship with the community PHE network, medical treatment, and rescue work in the community); (4) stockpiles of drugs and materials (stockpiles of drugs and other resources and personal protective equipment); (5)PHE detection and identification (syndrome surveillance); (6) procedures for medical treatment (protocol for diagnosis, treatment, and transfer of PHE victims); (7) laboratory diagnosis and management (laboratory regulation and management system, sample disposal and evaluation system, collection and disposal of suspected samples, and diagnosis of pathogen/etiology); (8) staff training (organization of PHE training, current training of medical staff, curriculum development and training effectiveness assessment); and (9) risk communication (organization for communication of risk psychological counseling to victim and medical staff, and communication with public). Excluding aspect 1, items 2–9 (covering 88 survey questions) represent 8 types of PHE preparedness capacities. Each answered item was scored 1 for "yes" and 0 for "no" or "unknown". Item scores were calculated by adding together "yes" answers. Items scores were used as a proxy for measuring PHE preparedness in an institution. A total item score was measured by calculating the score across all 8 items. The higher the total item score, the better the hospital PHE preparedness capacity. Further analyses were conducted to understand the correlation between preparedness capacity and demographic information. The distribution of the related preparedness capacities across 10 categories of PHE [1] and 15 types of etiology was also assessed.

### Data collection procedures

A computerized questionnaire stored in a CD was sent to the targeted hospitals accompanied by an official letter from each of the four city and provincial health departments stating the importance of the survey and requiring that each hospital designates a department director to be responsible for coordinating the completion of the questionnaire. Each returned questionnaire was carefully reviewed for its completeness and consistency. For those questionnaires with incomplete and/or inconsistent responses, one or two follow-up telephone calls were made to ensure completeness and consistency. The data from returned questionnaires were then transferred into a database for analysis.

### Data analysis

A database was set up using Microsoft Excel 2003. Data was checked, cleaned, and analyzed using SPSS software version 11.5. Ninety-five percent confidence interval of means (95% CI) was used to describe PHE preparedness capacities. Categorical variables were analyzed with frequency and percentage. Comparisons of mean score of each of eight PHE preparedness capacities among different types of hospitals were performed with P < 0.05 as statistical significance using parameter test (Independent-Samples T Test (two-tailed) or One-way Analysis of Variance) and/or non-parameter test (Mann-Whitney Test or Kruskal-Wallis Test) based on data distribution characteristics and homogeneity.

## Results

Four hundred hospitals responded, with a response rate of 100%. However, seventy-seven questionnaires were excluded from analysis due to one of the following reasons: (1) if less than 50% of items in the questionnaire were not answered, or (2) hospital did not meet secondary and/or tertiary hospital standard according to the hospital classification system. Therefore, the valid response rate was 79.5%.

### Hospital demographic information

Of analyzed hospitals (318), 29.9% were in Beijing, 24.5% in Shandong, 40.6% in Guangxi and 5.0% in Hainan. In terms of hospital type, 72.4% were teaching hospitals. The mean number of physicians and nurses per hospital was 174.5, and the mean number of total medical staff per hospital was 206.1. The mean number of physicians and nurses in emergency department and infectious-disease department were 24.3 and 12.0, respectively. Table 1 shows the demographic characteristics of the analyzed hospitals.

**Table 1:** Demographic characteristics of the surveyed hospital, Beijing, Shandong, Guangxi, and Hainan, China, 2004–2005 (N = 318)

Variables	Tertiary grade A^1^	Tertiary grade B^1^	Secondary grade A^1^	Secondary grade B^1^	Total^4^
Region					318
Beijing	34	3	53	5	95
Hainan	5	0	6	5	16
Shandong	16	10	38	14	78
Guangxi	20	8	91	10	129

Fever clinics					316
Yes, designated	50	17	119	25	211
Yes, not designated	4	1	29	3	37
No	20	3	39	6	68

SARS patients admitted^2^					313
Yes	25	5	38	0	68
No	48	16	147	34	245

Teaching hospitals					315
Yes	68	18	125	17	228
No	6	3	61	17	87

Types of hospital^3^					297
General hospital	41	12	117	19	189
TCM hospital	8	4	25	4	41
TCM-WM hospital	1	1	5	0	7
Specialized hospital	18	4	30	6	58
Community health center	0	0	1	0	1
Emergency center	1	0	0	0	1

^1^The hospital classification system, see in the study design section.

^2^SARS patient admitted means the status whether the hospital admitted SARS patients during SARS crisis in 2003.

^3^Types of hospital, see in the study design section.

^4^Some total number of hospitals may not be 318 due to the missing values.

### Hospital PHE preparation (Capacity 1)

Of 318 hospitals, 264(85.2%) had an emergency plan. Among the 264 hospitals that had an emergency plan, 92.6% reported that the institution possessed a protocol to initiate the emergency plan, 75.5% had a classification system for different PHE events, 55.3% had evaluated and revised their emergency plan at least once, and 79.6% reported that their emergency plan was accessible to all medical staff. As for organizational preparation, 93.3% of hospitals with an active emergency plan had a command center and designated personnel for PHE situations. There were statistical significance among tertiary grade A hospitals (95% CI: 9.8,10.9) and secondary grade B ones(95% CI:7.0,9.3), tertiary grade B hospitals(95% CI:9.8,11.9) and secondary grade B ones. Comparison of eight aspects PHE preparedness capacities among different types of hospitals are showed in table 2 and table 3.

**Table 2:** Comparison of eight aspects PHE preparedness capacities (capacity 1 to 4) among different characteristics of hospitals, Beijing, Guangxi, and Shandong, Hainan, China, 2004–2005 (N = 318)

Variables		Number	Capacity 1 95% CI	Capacity 2 95% CI	Capacity 3 95% CI	Capacity 4 95% CI
Region	Beijing	95	9.6,10.6	5.9,6.9*	5.8,6.9**	5.4,6.3
	Hainan	16	6.9,9.8	5.4,8.0*	4.8,7.8**	3.9,6.9
	Shandong	78	9.0,10.3	7.0,7.9*	6.6,7.9**	5.2,6.3
	Guangxi	129	9.2,10.2	6.7,7.4*	5.4,6.4**	5.4,6.2

Classification	Tertiary grade A	75	9.8,10.9**	6.8,8.0*	6.6,7.9**	5.6,6.6*
	Tertiary grade B	21	9.8,11.9**	6.7,8.4*	6.3,9.0**	5.6,7.3*
	Secondary grade A	188	9.3,10.1**	6.5,7.1*	5.8,6.6**	5.4,6.0*
	Secondary grade B	34	7.0,9.3**	5.4,6.9*	3.9,6.0**	4.1,5.9*

Teaching hospital	Yes	228	9.6,10.3**	7.0,7.5**	6.4,7.1	5.6,6.2
	No	87	8.7,9.9**	5.7,6.6**	5.0,6.1	5.1,6.0

Type	General hospital	189	9.7,10.5**	7.0,7.6	6.2,7.0	5.5,6.2
	TCM hospital	41	8.4,10.2**	5.5,6.9	5.1,6.8	5.3,6.7
	TCM-WM hospital	7	7.7,14.5**	5.6,9.8	5.1,11.7	6.0,8.3
	Specialized hospital	58	8.5,10.0**	5.8,6.8	5.2,6.8	4.7,6.0
	Community health center	1	--**	--	--	--
	Emergency center	1	--**	--	--	--
SARS patients admitted	Yes	68	9.8,10.9	6.3,7.4	6.0,7.3	5.3,6.4
	No	245	9.3,10.0	6.7,7.3	6.0,6.7	5.5,6.1

† *0.01 < p < 0.05, **p ≤ 0.01; 95% CI: 95% confidence interval of means.

‡ Capacity1: Hospital PHE preparation (highest score = 13); Capacity2: Response to PHE in community (highest score = 11); Capacity 3: Stockpiles of drugs and materials (highest score = 12); Capacity4: Detection and identification of PHE (highest score = 8).

§The post-hoc multiple significant comparison shows that: Capacity1 (Tertiary grade A vs Secondary grade B; Secondary grade B vs Tertiary grade B; Secondary grade A vs Secondary grade B; General hospital vs Specialized hospital); Capacity2 (Beijing vs Shandong; Beijing vs Guangxi; Tertiary grade A vs Secondary grade B; Secondary grade B vs Tertiary grade B); Capacity3 (Beijing vs Shandong; Tertiary grade A vs Secondary grade A; Tertiary grade A vs Secondary grade B; Tertiary grade B vs Secondary grade A; Tertiary grade B vs Secondary grade B; Secondary grade A vs Secondary grade B); Capacity4 (Tertiary grade A vs Secondary grade B; Tertiary grade B vs Secondary grade B).

**Table 3:** Comparison of eight aspects PHE preparedness capacities (capacity 5 to 8) among different characteristics of hospitals, Beijing, Shandong, Guangxi, and Hainan, China, 2004–2005 (N = 318)

Variables		Number	Capacity 5 95% CI	Capacity 6 95% CI	Capacity 7 95% CI	Capacity 8 95% CI
Region	Beijing	95	6.5,8.1**	4.0,4.9	5.7,6.6*	3.4,4.4**
	Hainan	16	5.4,9.9**	3.6,5.6	4.2,7.5*	2.2,5.3**
	Shandong	78	8.8,10.7**	4.4,5.4	5.9,7.0*	4.6,6.2**
	Guangxi	129	8.5,9.8**	4.0,4.6	5.1,6.0*	3.2,4.1**

Classification	Tertiary grade A	75	8.2,10.2*	4.6,5.7**	5.9,7.0	4.7,6.0**
	Tertiary grade B	21	8.6,11.4*	4.4,5.9**	4.9,7.1	4.2,6.9**
	Secondary grade A	188	8.0,9.2*	4.1,4.6**	5.6,6.2	3.2,4.0**
	Secondary grade B	34	5.5,8.3*	2.6,3.9**	4.3,6.1	2.8,4.8**

Teaching hospital	Yes	228	8.6,9.6*	4.4,5.0	5.8,6.4	4.0,4.8
	No	87	6.8,8.5*	3.6,4.4	5.2,6.2	3.1,4.3

Type	General hospital	189	8.7,9.9**	4.5,5.0	5.9,6.5	3.7,4.5
	TCM hospital	41	7.3,9.6**	3.7,4.7	4.6,6.2	2.9,4.7
	TCM-WM hospital	7	8.9,13.7**	1.6,7.0	3.6,8.7	1.9,8.7
	Specialized hospital	58	5.5,7.7**	3.4,4.4	5.0,6.4	3.9,5.5
	Community health center	1	--**	--	--	--
	Emergency center	1	--**	--	--	--

SARS patients admitted	Yes	68	7.6,9.6	4.3,5.3*	5.9,6.8	3.1,4.3
	No	245	8.2,9.3	4.2,4.7*	5.6,6.2	3.9,4.7

† *0.01 < p < 0.05, **p < 0.01; 95% CI: 95% confidence interval of means.

‡ Capacity 5: Procedures for medical treatment (highest score = 15); Capacity 6: Laboratory diagnosis and management (highest score = 9); Capacity 7: Staff training (highest score = 9); Capacity 8: Risk communication (highest score = 11).

§ The post-hoc multiple significant comparison shows that: Capacity5 (Beijing vs Shandong; Beijing vs Guangxi; Tertiary grade A vs Secondary grade B; Secondary grade B vs Tertiary grade B; Secondary grade A vs Secondary grade B; General hospital vs Specialized hospital; TCM hospital vs Specialized hospital; TCM-WM hospital vs Specialized hospital); Capacity6 (Tertiary grade A vs Secondary grade A; Tertiary grade A vs Secondary grade B; Tertiary grade B vs Secondary grade B); Capacity7 (Shandong vs Guangxi);Capacity8 (Beijing vs Shandong; Hainan vs Shandong; Guangxi vs Shandong; Tertiary grade A vs Secondary grade A; Secondary grade B vs Tertiary grade A; Tertiary grade B vs Secondary grade A; Tertiary grade B vs Secondary grade B).

### Response to PHE in community (Capacity 2)

Of all analyzed respondents, 64.2% were designated as the local emergency hospital for PHE patient admissions and 53.0% of them were the designated hospitals to provide medical rescue services during a national disaster. Of all analyzed respondents, 97.4% could promptly transport needed medical staff to the PHE field, 84.5% reported that they were prepared to respond to the needs of vulnerable people (including women, children, pregnant women and the disabled) during a PHE, however, only 49.8% had evaluated their ability to increase beds and equipment for PHE. When performing a PHE preparedness drill, 22.9% of respondents reported that they would invite relevant community organizations to participate. With regard to capacity comparison, the statistics test showed: the total item score of hospitals in Beijing(95% CI:5.9,6.9) was lower than that of hospitals in Shandong (95% CI:7.0,7.9) and Guangxi(95% CI:6.7,7.4); the score of teaching hospitals(95% CI:7.0,7.5) was higher than that of non-teaching hospitals(95% CI:5.7,6.6); and the score of tertiary grade A (95% CI:6.8,8.0) and B (95% CI:6.7,8.4) hospitals was higher than that of secondary grade B ones(95% CI:5.4,6.9), respectively. Among all types of hospitals, community health center scored highest on this aspect.

### Stockpiles of drugs and materials (Capacity 3)

Our results revealed that 53.1% of respondents had evaluated their stockpiles of drugs, and 61.5% had established a relationship with suppliers to provide emergency drug-supplies, however, only 43.2% had signed written contracts with suppliers. Of all analyzed respondents, 47.8% had drug-distribution plans, and 21.5% knew where the national or local pharmacy distribution centers were located. In regards to other medical materials, 80.1% had stockpiles of materials for responding to PHE. As for the stockpiles of drugs for infectious diseases, about 93.2%, 91.9% and 43.5% of responding hospitals had drug stockpiles for treating infectious diarrhea, influenza and botulismo toxin, respectively. When hospitals were compared on this item, statistical analysis showed that institutions in Beijing (95% CI:5.8,6.9) had a higher score than that of Shandong (95% CI:6.6,7.9). Tertiary hospitals generally had a higher score than secondary ones.

### PHE detection and identification (Capacity 4)

Among all the respondents, 55.5% reported that they had developed syndromic surveillance systems for certain diseases and 84.4% required that physicians on duty should report any abnormity to the hospital's presidents (the definition of abnormity see in appendix). Abnormity in admission diagnosis, routine microbiological tests, emergency room patients, and death with unknown causes were systematically monitored by 74.6% of institutions and 47.4% of hospitals shared their surveillance information with the local health authority. There were statistically significant differences between tertiary grade hospitals (Grade A 95% CI: 5.6,6.6; Grade B 95% CI: 5.6,7.3) and secondary grade B hospitals (95% CI:4.1,5.9) for this capacity, with tertiary hospitals scoring higher on their ability to detect and identify a PHE.

### Procedures for medical treatment (Capacity 5)

Physicians in 80.2% of the responding institutions reported being familiarized with the latest treatment protocol for a PHE, 92.8% could transfer PHE victims to corresponding medical agencies for appropriate treatment, and 98.0% could provide training on the protocol system. However, only 69.0% had specific procedures for patient transfer in a PHE. As for infectious disease treatment protocol, 80.1% had protocols for SARS, but only 37.3% for brucellosis. With regard to the capacity comparison between evaluated hospitals, statistical analysis revealed that hospitals in Shandong (95% CI:8.8,10.7) and Guangxi (95% CI:8.5,9.8) scored higher than those of Beijing (95% CI:6.5,8.1). Furthermore, TCM-WM hospitals (95% CI:8.9,13.7) scored higher than all other types of institutions. Tertiary grade hospitals (Grade A 95% CI:8.2,10.2; Grade B 95% CI: 8.6,11.4) and teaching hospitals (95% CI:8.6,9.6) had better score than secondary grade B (95% CI:5.5,8.3)and non-teaching institutions (95% CI:6.8,8.5), respectively.

### Laboratory diagnosis and management (Capacity 6)

We selected 15 kinds of infectious diseases/etiologies on which to assess laboratory diagnosis capacity, medical treatment procedures, and drug stockpile for infectious disease control. Our results showed that 59.0% of responding hospitals could isolate and identify salmonella and staphylococcus, but less than 5% reported that they could isolate and identify human H5N1 avian flu and SARS. The results are listed in table 4. As for the management of laboratory of results, 97.4% of respondents had strict laboratory operational regulations and 96.4% had personnel specially assigned to laboratory management. When faced with an emergency, 76.7% could promptly enlarge the capacity of sample disposal, while only 33.5% had protocols to collect suspected samples. Disposal and transportation of suspected samples capabilities were 33.5% and 32.6%, respectively, but once laboratories were contaminated, only 9.1% had alternatives. Statistical analysis showed that tertiary-grade A (95% CI:4.6,5.7) and B (95% CI:4.4,5.9) hospitals and hospitals with experience of SARS patients (95% CI:4.3,5.3) scored higher than those secondary-grade and without experience (95% CI:4.2,4.7), respectively.

**Table 4:** The laboratory diagnosis capacity, medical treatment procedures and drug stockpile for 15 types of etiology in respondent hospitals, Beijing, Shandong, Guangxi, Hainan, China, 2004–2005 (N = 318)

Varieties of etiology	Laboratory diagnosis capacity		Medical treatment procedures		Drug stockpile	
	
	No. "yes"	%	No. "yes"	%	No. "yes"	%
SARS	9	3.1	241	80.1	252	80.8
Plague	28	9.8	136	47.6	149	49.0
Cholera	145	49.3	175	60.8	194	63.6
Anthrax	49	16.9	110	38.6	140	46.4
Brucellosis	43	15.0	106	37.3	134	44.8
Meningococcal meningitis	98	33.6	203	68.4	237	77.2
Japanese encephalitis B	43	14.8	203	69.0	242	79.1
Influenza	43	14.6	234	78.8	285	91.9
Human H5N1 avian flu	6	2.1	197	67.0	182	60.1
Infectious diarrhea	77	60.4	225	77.1	288	93.2
Food poisoning of staphylococcus	172	58.7	187	64.7	259	84.6
Food poisoning of salmonella	174	59.6	184	63.7	255	83.3
Acute organophosphorus poisoning	111	37.8	226	76.6	272	88.3
Botulism toxin poisoning	24	8.3	126	44.5	131	43.5
Tetramine poisoning	18	6.3	162	56.1	162	53.6

### Staff training (Capacity 7)

Among all the respondents, 94.5% reported that they had a training program for the following medical staff: infection managers (56.3%); emergency department physicians and nurses (92.2%); and infectious disease ward physicians and nurses (71.8%). Staff training was supervised by a designated person in 82.3% of institutions and 65.8% had training curriculums, 66.5% of which was updated regularly. Effectiveness of PHE training was periodically assessed in 50.3% of respondents. For this capacity, statistical significance indicated that respondents in Shandong (95% CI:5.9,7.0) scored higher than participating institutions in Guangxi (95% CI:5.1,6.0).

### Risk communication (Capacity 8)

Of all respondents, 45.0% possessed expert panels to advise on PHE psychological counseling for medical staff and PHE victims. Medical staff in 12.1% of the analyzed hospitals were trained to assess the psychological impact of PHE. If a PHE occurred, participating hospitals could: counsel victims and their family members (43.6%); offer teaching materials (51.0%); access additional psychological consultants (19.3%). With regards to communication capabilities, 39.4% of hospitals reported a mass media communication protocol, 43.9% possessed a designated spokesperson to deliver PHE information to the public, and 30.8% possessed personnel specially assigned to communicate information to the media, public, and local governments. Statistical analysis showed that hospitals in Shandong (95% CI:4.6,6.2) scored higher than those in Beijing (95% CI:3.4,4.4), Guangxi (95% CI:3.2,4.1) and Hainan (95% CI:2.2,5.3) on this capacity, with tertiary-grade hospitals (Grade A 95% CI: 4.7,6.0; Grade B 95% CI: 4.2,6.9) reporting better score than secondary ones (Grade A 95% CI: 3.2,4.0; Grade B 95% CI: 2.8,4.8).

### Comparisons of various PHE contents

Five aspects of preparedness capacities and various PHE events were described in crosstab, as shown in table 5. Among all the respondents, 277 hospitals (95.5%) had emergency plans for infectious epidemics, and 50 ones (20.5%) for biochemical and nuclear terrorism. Evaluation on infectious epidemic was performed by 93.7%, the percentage was relatively lower for bio-terrorism and nuclear terrorism threats (30.5%), In regards to expert consultation, 28.2% had attended local agency meetings on regulation and revision of emergency plans for infectious epidemic control and 93.3% had the available expert list for consultation on infectious epidemic, however, for terrorism, the percentage was only 23.1%. Projects for admitting and treating infectious epidemic victims were common (71%), although only 13.3% of hospitals were involved in similar plans for dealing with bio-terror and nuclear threats.

**Table 5:** Comparisons of various PHE events and related capacities of all types of hospitals, Beijing, Shandong, Guangxi, and Hainan, China, 2004–2005 (N = 318)

Varieties of PHE	Emergency plans included		Assessing response to PHE		Attending regulation and revision of emergency plans in local agencies		Having expert lists for following situation		Having projects admitting and treating following victims	
	
	No. "yes"	%	No. "yes"	%	No. "yes"	%	No. "yes"	%	No. "yes"	%
Infectious diseases incidence	277	95.5	133	93.7	81	28.2	293	93.3	210	70.7
Unidentified population diseases	167	62.8	110	79.1	54	19.0	231	75.0	141	48.8
Mass food poisoning and water pollution	200	73.5	116	82.3	57	20.1	243	79.2	173	59.5
Mass occupational poisoning	125	49.2	75	56.8	45	16.0	166	56.5	100	35.3
Outbreak of nosocomial infection	162	62.8	104	77.0	56	19.9	222	73.8	143	49.8
Mass abnormal reaction or death resulted from drugs or vaccination	93	38.0	79	59.8	41	14.5	160	54.8	91	32.4
Incident of radioactive or poisonous material contamination	103	41.4	60	45.8	32	11.4	125	43.1	73	25.9
Biochemical and nuclear terrorism	50	20.5	40	30.5	25	8.9	66	23.1	37	13.3
Grave medicine accident	203	76.0	115	83.9	58	20.4	237	77.7	158	54.7
Natural disaster	173	67.8	97	70.8	49	17.4	214	71.1	138	48.6

## Discussion

Serious PHE concerns were raised in China during the 2003 SARS crisis when it became apparent that hospitals possessed poor emergency preparedness [7]. Even the up-coming 2008 Olympics Game in Beijing and the 5.12 Earthquake Disaster in China have dramatically evoked the awareness of PHE preparedness capacity for hospital. Based on the experience of the SARS pandemic, all hospitals should possess fundamental PHE programs, including preparedness of drugs, equipment, staff, emergency education and staff training [3,8,9], coordination with relevant community bodies [10], medical treatment [11], early detection and warning [12], laboratory diagnosis [13-15] and psychological intervention [8]. Since the SARS crisis, the central Chinese government has become more active in the construction of public health system, especially in regards to the medical emergency response system [16]. One major effort involved a 11.4 billion RMB investment in local governments to initiate the construction of regional PHE medical treatment systems [17]. In order to offer some insight into the development of hospital PHE preparedness capacity, this study examined the current status of hospital preparedness in Beijing, Shandong, Guangxi, and Hainan.

Emergency preparedness refers to the processes involved in ensuring an institution: (1) has complied with the preventive measures; (2) is in a state of readiness to contain the effects of a forecasted disastrous event in order to minimize loss of life, injury, and damage to property; (3) can provide rescue, relief, rehabilitation, and other services in the aftermath of the disaster; and (4) holds the capability and resources to continue to sustain its essential functions during a PHE [18]. An emergency preparedness systems primarily composed of emergency plans and organizational structures and lays the foundation for dealing with PHE [19]. Emergency plans establish the protocol for operation under a PHE [16]. For a hospital to mobilize all PHE resources in a short period of time, contingency plans must be issued in advance [9]. In addition, periodic review and updating of emergency plans enhance an institution's emergency response capacity [3]. Our study showed that most hospitals had emergency plans and that these plans focused on infectious diseases control with less attention to preparedness for biological, nuclear radiation and other terrorism attacks. Most of the hospitals had PHE command departments and emergency response teams, however, only 55.3% of hospitals with emergency plans reported they had evaluated and revised their PHE systems. Overall, tertiary hospitals performed better in PHE preparation than secondary hospitals. Meanwhile, no statistical significance was found between hospitals that had admitted SARS patients and those that had not, suggesting that after the SARS crisis, all hospitals raised awareness of emergency plans and implementation.

No hospital or medical system can manage a public health emergency without community networks and public involvement. Therefore, hospitals need to communicate and cooperate with other local health agencies, functioning as a networked public health provider. Problems like lack of communication and coordination between hospital departments and inter-agency networks hinder the availability of resources in a community and limit timely forecasting, public communication and effective regulation of a PHE [10]. Our survey revealed that if a PHE occurred, most of hospitals reported that they could take responsibility for PHE rescue service, transport the medical staff in a timely manner, and provide priority health services to vulnerable populations. Yet, less than one third of respondents attended regulation and revision workshops for emergency plans for infectious epidemic control held by local agencies. This lack of cross-institutional interaction indicated that the ability of hospitals to coordinate with community agencies in preparation for, or in the event of a PHE was generally poor. The survey showed that among all the types of respondents community health center were best able to respond to PHE and the respondents with multiple functions performed better suggesting that communication and coordination between hospitals and community agencies should be strengthened.

Characteristics of a PHE include suddenness and unpredictability [9]. For most hospitals, medicine storage may be in great demand when faced with a sudden increase in patients. Therefore, hospitals must have programs to ensure appropriate levels of emergency supplies including drugs, medical equipment, electricity, water and oxygen, disinfectant, etc. Our survey suggested that most of the hospitals could establish an emergency-drug-supply system for most of the infectious diseases we addressed in the questionnaire except anthrax, brucellosis, botulism toxin poisoning and tetramine poisoning. For most of surveyed hospitals possessed emergency resource reserves, but less than half of them had corresponding drug distribution programs. In addition, hospital capacity was affected by economic level and classification of the hospital, suggesting that the importance of local economic development strengthens hospital ability to provide PHE.

Early detection and identification of a PHE are amongst the most important objectives for prompt and effective public health response to a PHE [12] as well as an essential precondition for selecting appropriate prevention and treatment measures. This study showed that most of the hospitals could regularly train medical staff on how to report and identify suspicious PHE and that the institutions possessed surveillance systems to monitor various aspects of abnormity. Approximately half of the respondents could share surveillance information with the local health authorities. There were statistically significant differences among various classification of the respondents, which demonstrated that after the SARS crisis, hospitals at all levels attached high importance to PHE monitoring and early warning system, however, the capacity was affected by the comprehensive strength of hospital.

PHE happens suddenly and its incidence rate is relatively low, which leaves most medical staff inexperienced and unprepared [11]. Therefore, it is important that hospitals develop emergency plans for PHE treatment programs. In this survey, more than half of respondents showed that their physicians were aware of current PHE protocols. Most hospitals had transfer and treating procedures for infectious diseases, including SARS, influenza, and infectious diarrhea, but less held these procedures for biochemical incidents, leakage of nuclear, and terrorist attacks. Because they are easily used as biological terrorist attacks materials [20], therefore, the prevention and control of these emergencies become very important. Our statistical analyses showed that tertiary-grade, teaching and TCM-WM hospitals performed better on medical treatment procedures preparedness, which might reflect the fact that different types of hospitals have different functions and mission in the community, however, for this capacity, the statistical significance among different regions showed the important role that economic factor plays.

Hospital laboratories not only have the task of clinical diagnosis, but take some responsibility in the surveillance of public health [13,14]. Therefore, laboratory information plays an important role in detection of the PHE [13,15]. Detecting PHE related pathogen/etiology can not only confirm clinical diagnosis, but also identify newly emerging infectious diseases [15,21]. The presence of SARS in China in 2003, and the slow response to its emergence, revealed that China's public health laboratory systems were weak [13]. This survey indicated that many of the hospitals did not report adequate laboratory diagnostic capacities. Although hospital laboratory regulations seemed relatively good, only one-third of hospital laboratories had programs for dealing with suspicious samples collecting, disposal and delivery. Only half of the surveyed hospitals could detect food-borne pathogens, including cholera vibrio, infectious diarrhea, staphylococcus and salmonella. Few hospitals had the capacity to detect the airborne pathogens, including brucella, influenza virus, anthrax bacteria, the H5N1 avian flu virus, plague bacillus, meningitis B virus, SARS virus and other pathogens. Hospitals with admitting SARS patients performed better showed the importance of the experience of SARS disposal.

Prior to the 2003 SARS crisis, China had not experiences a large-scale PHE outbreak for some time. As PHE is a high-risk event with little probability [22], medical staff often possess limited awareness of appropriate response and this contributed to the under-detection of SARS nosocomial infections in 2003 [23]. When PHE occurs, hospital medical staff are usually the first responders and information providers, therefore, education and training are key measures to enhance PHE response [24]. Our survey suggested that after SARS crisis, most hospitals re-evaluated the importance of medical staff training for PHE. The majority of respondents offered training programs to their related medical staff. However, the effectiveness of these training programs needs to be periodically evaluated.

PHE can cause psychological as well as physical problems for the public and medical staff attending to victims [9,25]. In a public health crisis or emergency, effective risk communication can help people cope, make decisions, and return their lives to normal. Crisis communication, as an important part of a PHE response [8], is key to ensuring complete, transparent and prompt information exchange, and to help hospitals make timely responses and reduce the serious consequences [26]. The results of this survey revealed that medical staff in 12.1% of the hospitals underwent training for evaluation of PHE-related stress and only one-third of respondents had specific programs and spokespersons for communicating critical messages and information to the media, public, governments and stakeholders. These results indicated that most of the surveyed hospitals do not understand the importance of psychological care in a PHE emergency, do not have the resources to deal with it, or presume that it is not their place to do so. Indeed, this capacity evaluation revealed that when a PHE occurred, most hospitals' response plans focused on physiological medical treatment, but health education, psychological counseling, and crisis communication plans were rare. However, for this capacity, the statistical significance among different regions and levels showed the important role that economic factor and comprehensive level play.

### Limitations

The study has several limitations. First of all, the surveyed hospitals were restricted to four city and provinces, even some types of hospitals were rare (the number of the surveyed community health center and emergency center was just one, respectively), therefore, the results may not fully represent the PHE capacity of all hospitals in China. Secondly, because of self-report method there may be a respondent reporting bias. The inclusion of official documents from respective Health Bureaus, for example, may have encouraged respondents to complete survey but have also been interpreted as an official assessment of capacity leading some hospital representatives to overestimate PHE capacity. Thirdly, only quantitative data were collected to measure certain capacities of PHE preparedness. Most questions required a "yes" "no" or "unknown" answer which restricts the collated data to these three categories. Finally, this data set is not complete as some hospitals did not respond and others had to be excluded on the basis of incomplete answers or for ineligibility for hospital classification. To a certain extent, this loss of respondents caused a loss of information.

## Conclusion

After several years of construction and development, the capability of hospitals in China to deal with PHE, in particular infectious diseases control, has improved greatly [3,4]. Nevertheless, this research suggests that China has more progress to make before PHE preparedness is satisfactory. To enhance hospital preparation for dealing with PHE, governments at all levels should increase investment in the construction of infrastructure to create and sustain appropriate PHE capacity. On the other hand, hospitals at all levels should enhance their management, including updating and revising of emergency plans; strengthening communication and cooperation with other local agencies; enhancing the capacity of abnormity monitoring and laboratory diagnostic capability for infectious diseases; improving the treatment program for various PHE scenarios; and strengthening psychological intervention and risk communication capabilities. Finally PHE preparedness in relation to terrorism caused by nuclear radiation and bio-chemical substance was low in this study and should be further assessed for areas of need and improvement.

## Appendix

Abnormity: Abnormity means the rapid increase of emergency room patients with acute asthma, flu, fever of unknown causes.

Hospital classification: According to "the hospital classification system" of the Ministry of Health of People's Republic of China, all hospitals in China are classified into primary, secondary, and tertiary hospitals based on their functions in providing medical care, medical education, and conducting medical research. A secondary hospital is defined as a regional hospital that provides comprehensive medical care, medical education, and medical research for the region. A tertiary hospital is defined as cross-regional, providing comprehensive and specialized medical care with a high level of medical education and research functions. Secondary and tertiary hospitals are further classified into subgroups: Grade A, Grade B, and Grade C according to their service levels, size, medical technology, medical equipment, and management and medical quality [27].

Community health center: community health center is a kind of primary health care delivery in China, most of which are transferred from secondary grade hospitals, and provide preventive, curative care, maternal and child care, rehabilitation and health education to local inhabitants by general practitioners, community nurses and public health workers.

Medical emergency center: medical emergency center (First Aid Station) is a kind of emergency health care delivery in China, which provide emergency care, first aid, monitoring and treatment for all those patients with pre-hospital emergencies.

## Competing interests

The authors declare that they have no competing interests.

## Authors' contributions

JSH designed the study and developed the tools. HZH participated in design of the study and development of the tools, and supervised the data collection and data entry. XML performed data checkup, data analysis and drafted the manuscript. All authors participated in discussion, revision and approved of the final manuscript.

## Pre-publication history

The pre-publication history for this paper can be accessed here:


